# Editorial: Genetics and mechanisms of neurodevelopmental disorders

**DOI:** 10.3389/fnhum.2026.1829859

**Published:** 2026-04-02

**Authors:** Anjana Munshi, Ajay Kumar, Santasree Banerjee

**Affiliations:** 1 Department of Human Genetics and Molecular Medicine, School of Health Sciences, Central University of Punjab, Bathinda, India; 2 Department of Genetics, College of Basic Medical Sciences, Jilin University, Changchun, China

**Keywords:** genetic variants, genotype-phenotype correlation, molecular mechanisms, neurodevelopmental disorders, novel mutations, whole-exome sequencing

Neurodevelopmental disorders (NDDs) are characterized by the failure of children to attain expected cognitive, emotional, and motor developmental milestones. These disorders arise from disruptions in the precisely coordinated processes that govern brain development. NDDs represent a significant public health issue, affecting over 3% of children globally (Gilissen et al., 2014). They are etiologically diverse and frequently result in impairments in cognition, communication, adaptive functioning, and psychomotor abilities. The spectrum of NDDs includes autism spectrum disorder, intellectual disability, attention-deficit/hyperactivity disorder, and other epilepsies (Niemi et al., 2018; Tǎrlungeanu and Novarino, 2018). Recent evidence indicates that shared molecular mechanisms may underlie the varied clinical manifestations of NDDs (Cristino et al., 2014; Hormozdiari et al., 2015), which accounts for the common co-occurrence of conditions such as intellectual disability, autism, and epilepsy within the same individual (Van Bokhoven, 2011; Du et al., 2018). Among these, autism spectrum disorder (ASD) continues to be one of the most thoroughly researched but mechanistically intricate conditions (Hodges et al., 2020). While significant advancements have been achieved in pinpointing genetic factors, the biological mechanisms connecting genetic predisposition to modified neurodevelopment are still not fully elucidated.

The Research Topic, “*Genetics and mechanisms of neurodevelopmental disorders*,” is highlighting recent advancements in genetic etiologies, evaluating molecular mechanisms, and developing better diagnostic methods for neurodevelopmental disorders. Based on a thorough peer review, 12 out of 20 submissions were accepted, indicating that it is a hot area of research currently. The accepted articles encompass clinical genetic studies, case reports, methodological advancements, and analyses of genotype–phenotype correlations that collectively enhance our comprehension of the genetic architecture of neurodevelopmental disorders (Bagatelas et al.; Chen et al.; Ge et al.; Guerrera et al.; Jiang, Xu et al.; Jiang, Li et al.; Li et al.; Li and Chen; Napoli et al.; Wang et al.; Wu et al.; Yan et al.).

Numerous studies in this compilation concentrated on discovering novel genetic variants and clinically delineating rare neurodevelopmental syndromes. Chen et al. published a full clinical and genetic study of ERCC8-related Cockayne Syndrome. They found that hepatic dysfunction could be a biomarker and that anhidrosis is a rare clinical feature. The authors also looked into the results of rehabilitation for ankle contractures, which gave them useful information about how to treat people who have them (Chen et al.). Expanding the spectrum of structural genomic variations associated with neurological diseases, Jiang, Li et al. identified a novel 9q21.13 microdeletion in a family presenting with epilepsy, intellectual disability, and speech impairment, emphasizing the importance of CNV analysis in patients with unexplained neurodevelopmental phenotypes (Jiang, Li et al.). Chromosomal deletions affecting key developmental genes represent another important mechanism underlying these disorders. In this context, Ge et al. analyzed cases with terminal 6q deletions and demonstrated that *DLL1* haploinsufficiency contributes to prenatal brain anomalies in these patients. Their study highlights the role of genes involved in NOTCH signaling in early brain development and highlights the importance of prenatal genetic diagnoses for structural brain abnormalities to avert the birth of children with brain anomalies (Ge et al.).

In addition to central nervous system disorders, some studies have provided insights into neuromuscular diseases that share overlapping developmental and genetic mechanisms. Li et al. reported compound heterozygous variants in *DYSF* in a family affected by limb-girdle muscular dystrophy type 2B, expanding the mutational spectrum of this disorder and contributing to better genetic diagnosis and improved patient management (Li et al.).

ASD is one of the most genetically heterogeneous neurodevelopmental conditions. Guerrera et al. evaluated the genetic profile of autistic children and adolescents bearing minimal verbal abilities, a subgroup that often faces diagnostic as well as therapeutic challenges. Their findings portray the genetic architecture of severe ASD phenotypes and highlights the significance of integrating genomic analysis into clinical settings (Guerrera et al.).

In consensus with this “Research Topic,” Wu et al. created and tested a phenotype-driven predictive model to figure out how trio-based whole-exome sequencing (trio-WES) helps in diagnosing of neurodevelopmental disorders in children with a genetic components. Such predictive frameworks may assist clinicians in identifying suitable patients for genetic testing and improving diagnostic methodologies in clinical practice. Yan et al., based on their research assessing clinical features, diagnosis, treatment, and prognosis of atezolizumab-induced encephalitis and aseptic meningitis, emphasized the necessity for clinical awareness for immune-related neurological adverse events associated with immunotherapy (Yan et al.).

Bagatelas et al. conducted further investigations into genotype–phenotype correlations. The authors showed that the main clinical signs of Okur-Chung Neurodevelopmental Syndrome (OCNDS) stay the same across different CSNK2A1 variants. Nonetheless, mutations situated in conserved loop regions of CK2α, especially the glycine-rich loop, correlated with heightened neurological symptomatology, an elevated incidence of hypotonia, and an expedited diagnosis. These findings yield significant insights into the structural and functional ramifications of these pathogenic variants (Bagatelas et al.).

Wang et al. used the FAERS database to carry out a signal-mining analysis of bad events in association with botulinum toxin type A. This has implications for its clinical use in patients affected with cerebral palsy (Wang et al.). The study has addressed therapeutic safety or in other words pharmacovigilance aspects.

Few case reports included in this issue have enriched the concept that the brain developmental gets affected on account of genetic variants. Jiang, Xu et al. identified biallelic variants in the MRPS36 gene associated with Leigh syndrome, a severe mitochondrial disorder characterized by progressive neurodegeneration. The results of their study added to our understanding of the function of mitochondrial ribosomal proteins in neurodevelopmental disorders (Jiang, Xu et al.). Another study by Napoli et al., studied a complex neurodevelopmental syndrome and identified variants of MECP2 and GABBR1 genes, in association with the same. This highlights as to how concurrent genetic modifications lead to atypical phenotypes and developmental pathways (Napoli et al.). A novel splice-site variant in SSR4 gene involved in congenital disorder of glycosylation type Iy, was identified by Li and Chen, thereby broadening the mutation spectrum of glycosylation disorders impacting neurological development (Li and Chen).

In conclusion, this current edition “Research Topic” adds to the evolving field of neurodevelopmental genetics, illustrating the significant role of genomic technologies in clinical diagnosis and research and thereby, devising novel therapeutic interventions based on the functional implications of the significant biomarkers.
